# Effect of preoperative topical diclofenac on intraocular interleukin-12 concentration and macular edema after cataract surgery in patients with diabetic retinopathy: a randomized controlled trial

**DOI:** 10.3325/cmj.2017.58.49

**Published:** 2017-02

**Authors:** Aleksej Medić, Tomislav Jukić, Anita Matas, Katarina Vukojević, Ada Sapunar, Ljubo Znaor

**Affiliations:** 1 Eye Clinic Medić Jukić, Split, Croatia; 2 Department of Ophthalmology, University Hospital Center Zagreb, Zagreb, Croatia; 3 Department of Ophthalmology, University Hospital Center Split, Split, Croatia; 4 Laboratory for Early Human Development, Department of Anatomy, Histology and Embryology, University of Split School of Medicine, Split, Croatia; 5 Medical Diagnostic Laboratory, University Hospital Center Split, Split, Croatia

## Abstract

**Aim:**

To determine if preoperative treatment with a topical non-steroidal anti-inflammatory drug (NSAID) lowers the concentration of intraocular interleukin (IL)-12 and the incidence of postoperative macular edema in patients with non-proliferative diabetic retinopathy undergoing cataract surgery.

**Methods:**

A total of 55 patients were randomized to diclofenac (n = 27) or placebo (n = 28). Patients receiving diclofenac started preoperative treatment with 0.1% topical diclofenac four times a day 7 days before cataract surgery and the therapy was discontinued 30 days after surgery. Patients in the control group were administered placebo 7 days preoperatively and a standard postoperative therapy with 0.1% topical dexamethasone four times a day for 30 days after surgery. All patients received postoperative antibiotic prophylaxis with tobramycin eye drops four times daily for 30 days. Seven days before the cataract surgery, on the day of surgery, and 1, 7, 30, and 90 days after surgery, central foveal thickness (CFT) was measured with optical coherence tomography (OCT) and the aqueous humor was sampled at the beginning of cataract surgery for the analysis of IL-12 concentration. Due to loss to follow-up and insufficient aqueous humor samples, the data of 3 patients treated with diclofenac and 8 patients receiving placebo were not analyzed.

**Results:**

The aqueous humor IL-12 concentration was significantly lower in the diclofenac group than in the placebo group (*t*=−2.85, *P* = 0.007). The diclofenac group had a significantly smaller increase in CFT after phacoemulsification (F = 13.57, *p*<0.001).

**Conclusion:**

Patients preoperatively treated with diclofenac had significantly lower intraocular levels of IL-12 and a lower increase in CFT, which indicates that a combination of preoperative and postoperative treatment with a topical NSAID may lower the incidence of postoperative macular edema in patients with diabetic retinopathy.

**ClinicalTrials.gov trial registration number:**

MZJ-2106

In the USA alone, approximately 29,1 million people (9.3% of the population) suffer from diabetes mellitus (1). The prevalence of diabetes mellitus in Croatia is 6.1% in the 18-64-year age group (2). It is estimated that diabetic retinopathy, one of major diabetes complications, is among the leading causes of vision loss in adults aged 20–74 years (3). Diabetes also accelerates the formation of visually significant cataracts (4), and diabetic patients often benefit from cataract surgery in both eyes (5). However, several studies have suggested that cataract surgery, which is usually a definitive treatment for this type of visual impairment, worsens the underlying diabetic retinopathy and macular edema (6,7). The leakage of the intravascular contents from dilated perifoveal capillaries initially causes thickening of the macula (edema formation), which may over time progress to cystoid space formation within the outer plexiform layer and inner nuclear layer of the retina, leading to cystoid macula edema (CME) (8). Several authors have found significantly increased intraocular concentrations of proinflammatory interleukins IL-6, IL-8, and IL-12 in patients with diabetes compared with patients without diabetes (9-11). IL-12 plays an important role in the enhancement of the cytotoxic activity of NK cells and CD8+ cytotoxic and T-lymphocytes (11). IL-12 is secreted by human dendritic cells and macrophages in response to various signals associated with defense and wound healing (11). Involvement of proinflammatory interleukins indicates that some patients with diabetic retinopathy have a subclinical intraocular inflammation. Patients with diabetes undergoing phacoemulsification cataract surgery seem to have a doubling of diabetic retinopathy progression rates 12 months after surgery (12). A significant thickening of the retina in the macular region was found in a recent study comparing the postoperative changes in central foveal thickness (CFT) measured by optical coherence tomography (OCT) in patients with diabetes and healthy subjects treated for cataracts (13). Modern cataract surgical techniques, characterized by small incisions and short operating times, have little influence on the progression of diabetic retinopathy, except on developing macular edema (14). Despite advances in cataract surgery, cystoid macular edema is still recognized as one of the most common causes of poor visual outcome following cataract surgery (15).

Two types of compounds are available for topical use to reduce the risk of CME after cataract surgery: corticosteroids and nonsteroidal anti-inflammatory drugs (NSAIDs). Corticosteroids are effective in suppressing postoperative inflammation, but have little suppression effects on CME and potentially increase intraocular pressure. Several trials in the effectiveness of NSAIDs in the treatment of CME following cataract surgery showed positive effect of NSAID-s on postoperative macular edema (16-19). A few studies investigated that effect in patients that started NSAID therapy preoperatively (20,21), and we found only one study in adult diabetic patients with non-proliferative diabetic retinopathy requiring cataract surgery that instilled NSAID therapy before surgery (22).

Diclofenac inhibits cyclooxygenase, an enzyme essential in the biosynthesis of prostaglandins. It is indicated for the treatment of postoperative inflammation in patients who have undergone cataract extraction and for the temporary relief of pain and photophobia in patients undergoing corneal refractive surgery.

We reviewed the available literature and found that, in patients with diabetes, the efficacy of both preoperative and postoperative topical diclofenac administration aimed at postoperative macular edema incidence reduction has not been explored. Additionally, quantitative analysis of postoperative CME with cataract surgery in patients preoperatively treated with topical diclofenac has not been studied. Therefore, our aim was to examine the effect of preoperative topical diclofenac treatment on lowering intraocular IL-12 concentration and macular edema formation following phacoemulsification in patients with diabetic retinopathy.

## PATIENTS AND METHODS

The prospective, double-blind, randomized placebo-controlled clinical trial was performed between January 2013 and December 2014 in collaboration between the Eye Clinic Medić Jukić, Split, Croatia, and the Clinical Hospital Center Split, Croatia, in accordance with the Declaration of Helsinki. Patients included in this study received both written and oral information about the study protocol and signed a written informed consent.

### Patients

**Figure 1:**
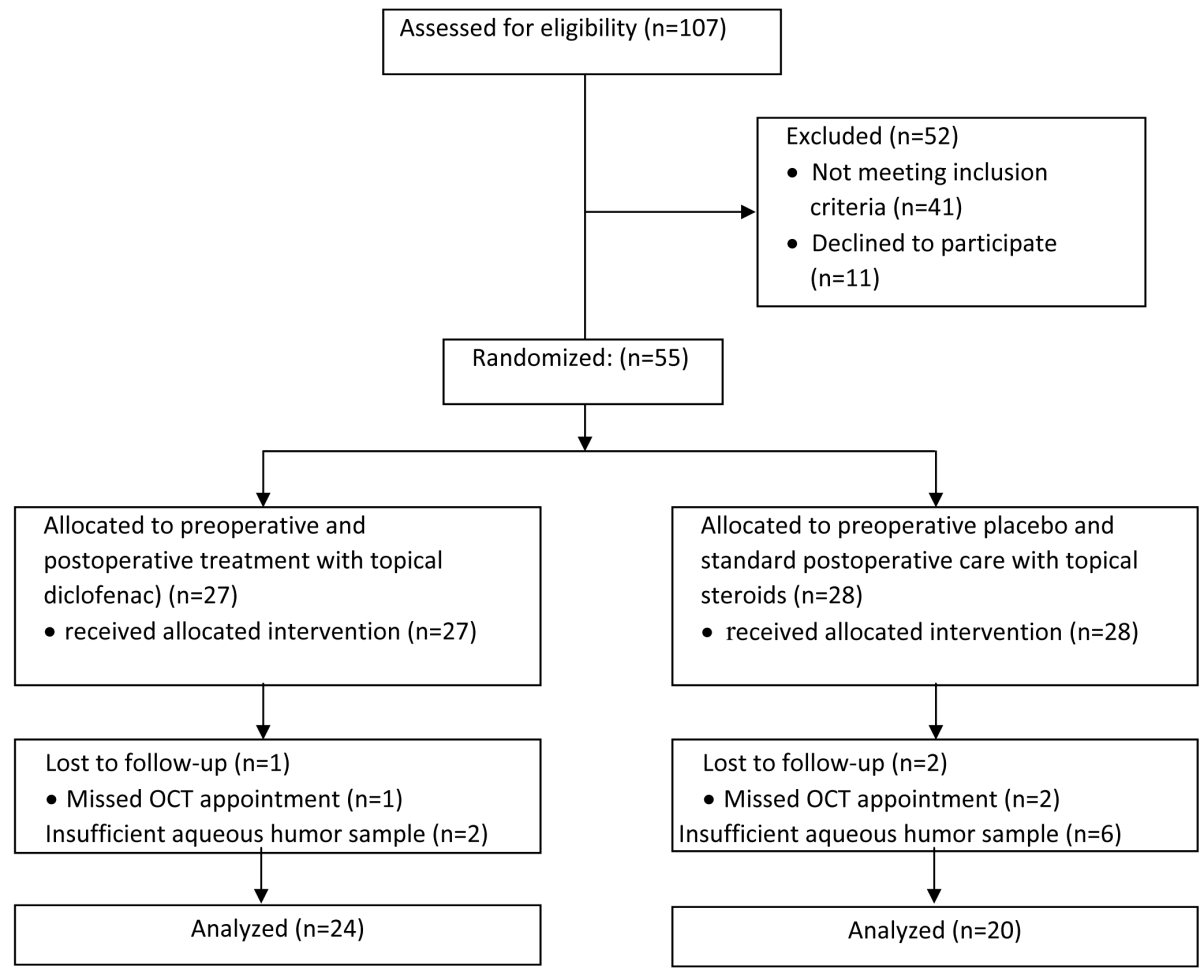
Flow diagram of patients included in the study.

Between January 2013 and June 2014, 107 diabetic patients referred for cataract surgery were assessed for eligibility (Figure 1). Inclusion criteria were age over 60 years, presence of mild and moderate diabetic retinopathy classified in accordance with Early Treatment Diabetic Retinopathy Study (23), and presence of cataracts graded according to the Lens Opacities Classification System, version III (LOCS III) as grade 2-3 (24). We did not include patients with other chronic or acute eye diseases (uveitis, central or branch retinal vein occlusion, or presence of epiretinal membrane), previous laser photocoagulation, and hypersensitivity to any component of the diclofenac eye drops, and those on oral anticoagulant therapy or allergic to salicylates.

Patients with diabetic retinopathy included in the study were randomized using sealed envelopes into either the experimental group receiving topical diclofenac or the control group receiving placebo. Topical treatment was prepared in unlabeled bottles, so that the patients and all examiners were blinded for the type of treatment applied.

### Methods

Patients who met all inclusion criteria were invited to participate in the study and were examined at one study site. At the initial visit, the patients signed informed consent form and underwent a complete ophthalmologic examination performed by a single examiner.

Complete eye examination included best corrected visual acuity, Goldmann applanation tonometry, slit lamp biomicroscopy of the anterior eye segment, binocular indirect slit lamp fundoscopy, fundus photography, and spectral OCT of the macular area (SOCT Copernicus, OPTPOL Technology, Zawiercie, Poland). Ophthalmologic examination was performed 7 days before the cataract surgery, on the day of surgery, and on days 1, 7, 30, and 90 after surgery.

In the experimental group, patients started the preoperative treatment with 0.1% topical diclofenac four times a day (Naclof, Exelsion, France) 7 days before the cataract surgery, and the therapy was discontinued 30 days after surgery. In the control group, patients were administered placebo 7 days preoperatively and the standard postoperative therapy with 0.1% topical dexamethasone (Maxidex Alcon, USA) four times a day for 30 days after surgery. All patients received postoperative antibiotic prophylaxis with tobramycin eye drops (Tobrex, Alcon, USA) administered four times daily for 30 days. Upon the start of cataract surgery, 0.2 mL of the aqueous humor was taken for IL-12 concentration measurements by enzyme-linked immunosorbent assay using an EASIA ELISA kit (KAC1561, BioSource Inc., USA). Quantitative analysis of IL-12 was performed at the Department for Medical Laboratory Diagnostics of the Split University Hospital Center, Split, Croatia. Cataract surgery was done with phacoemulsification (Alcon Infinity Phaco Machine, Alcon Inc., Hünenberg, Switzerland).

### Statistical analysis

Descriptive and inferential statistical analyses were performed. The parameters for diabetes duration and IL-12 concentration were expressed as arithmetic mean with standard deviation (mean±SD), and for phacoemulsification time, diabetes duration, and age as median with range. Frequencies were used in the description of the clinical profile (CME and worsening of diabetic retinopathy). Student’s *t*-test was used to test the mean difference between groups for IL-12 concentrations and phacoemulsification time, χ^2^ test was used to test the differences in cystoid macular edema frequency, and ANOVA for repeated measurements was used to analyze central foveal thickness change during follow-up visits. The difference in concentrations of IL-12 in the aqueous humor was examined with the unpaired *t*-test. The correlation of preoperative intraocular inflammation (IL-12 concentration in the aqueous humor) and postoperative changes in the CFT was examined with Pearson correlation analysis and at each time point separately (0, 1, 7, 30, and 90 days postoperatively). Normal distribution of the data was checked by Kolmogorov-Smirnoff test. For all tests, the significance level was set at *P* = 0.05. The study was powered enough to detect a mean difference in IL-12 concentrations of 33.2 pg/mL between the groups, under the assumptions of SD in the experimental and control groups of 26.5 and 29.9, respectively, two-sided significance level of 0.05, power of 80%, and at least 14 patients allocated in experimantal and 12 in the control group. Data were analyzed with SPSS 13.0 software (SPSS Inc., Chicago, Illinois, USA).

## RESULTS

**Figure 2:**
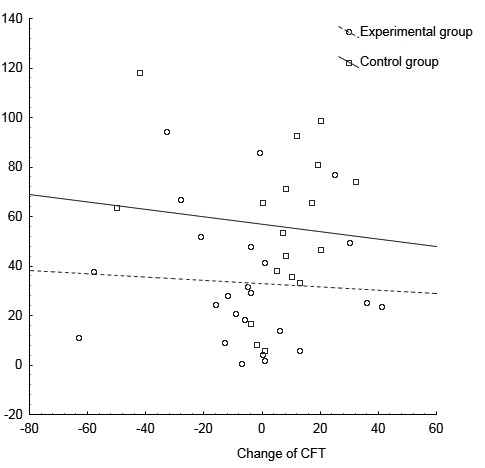
Correlation between interleukin (IL)-12 concentrations in humor aqueous and the change of central foveal thickness (CFT) between day 7 before surgery and the day of surgery when the aqueous humor was sampled in patients receiving diclofenac (circles) and placebo (squares).

**Figure 3:**
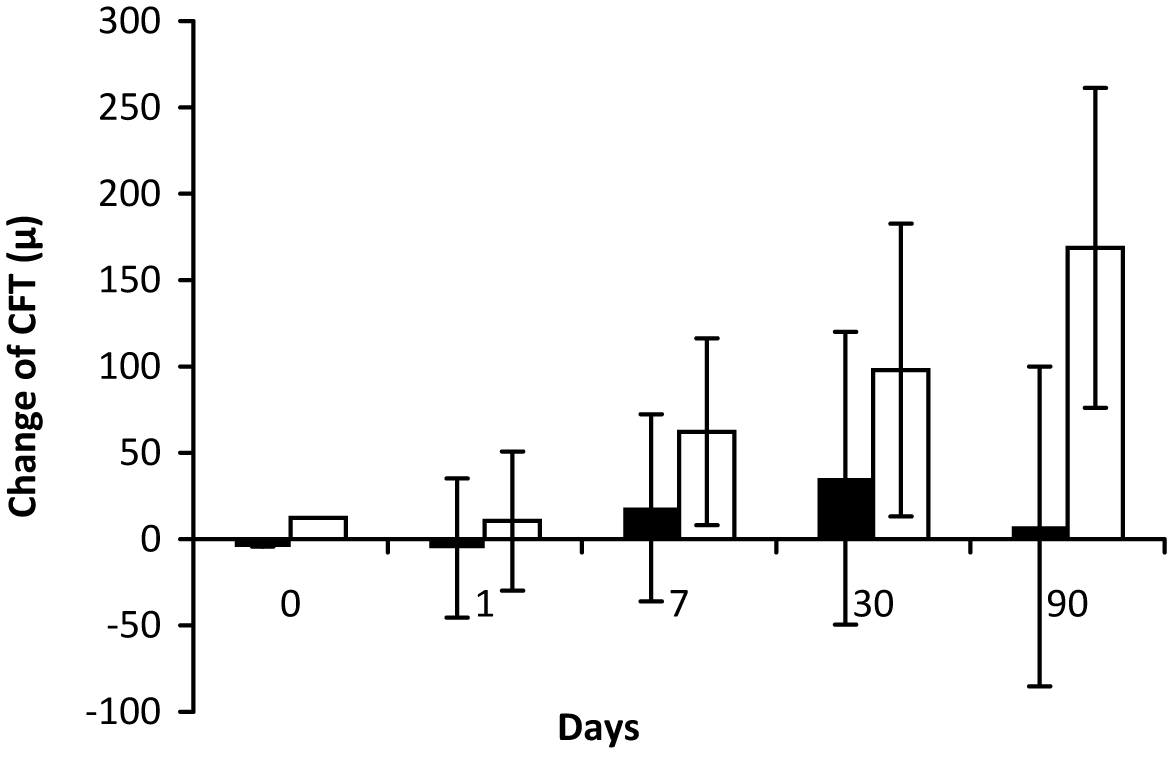
Mean change of the central foveal thickness (CFT) in the experimental group treated with diclofenac for 7 days before surgery (full bars) and the control group receiving placebo (empty bars). The change was calculated as the difference in CFT between baseline measurement 7 days before the phacoemulsification and each control visit on the day of surgery (day 0) and 1, 7, 30, and 90 days after surgery. Boxes and whiskers represent mean valued and standard deviations.

This study included 55 patients with type 2 diabetes (28 men, 27 women), with a mean age of 64.5 ± 17.5 years and a mean diabetes duration of 20 ± 15 years (Table 1). The experimental and control groups did not differ significantly according to age, sex, and the time from diagnosis of diabetes. Due to patient loss-to-follow-up and insufficient aqueous humor samples, the data of 3 patients in the experimental group receiving diclofenac and 8 patients in the control group receiving placebo were not analyzed (Figure 1). The aqueous humor IL-12 concentration, as an indicator of intraocular inflammation, was significantly lower in the experimental group (33.4 ± 26.5 pg/mL) treated preoperatively with diclofenac than in the control group receiving placebo (57.7 ± 29.9 pg/mL; *t* = –2.85, *P* = 0.007). There was no significant correlation between the CFT change and the IL-12 concentration in the aqueous humor on the day of the surgery in either group (Figure 2). Patients in the experimental group had a significantly smaller increase in CFT after phacoemulsification compared to patients in the control group (F = 13.57, *P*<0.001; Figure 3). In the experimental group, the peak of change in CFT was recorded at day 30 after surgery with subsequent decrease toward the day 90 postoperatively. In the control group, the CFT change progressively increased peaking on day 90 after surgery. The phacoemulsification time in the control and experimental groups did not differ significantly (Table 1). During the follow-up period, the OCT scans showed cystoid macular edema (CME) in 10 (37%) patients in experimental group and 12 (43%) patients in the control group (χ^2^ test = 87, *P* = 0.87).

**Table 1:** Demographic characteristics, duration of diabetes, and the duration of the phacoemulsification time in patients with diabetic retinopathy receiving diclofenac and placebo

	No. (%) of patients		
**Characteristics**	diclofenac group (n = 27)	placebo group (n = 28)	P*
Age (median, range; years)	73 (59–81)	70 (47–82)	
Sex			
women	13 (48.1)	14 (50.0)	
men	14 (51.9)	14 (50.0)	
Duration of diabetes (median, range; years)	18.9 (5.0-35.0)	16.7 (10.0-28.0)	0.420
Phacoemulsification time (median, range; cde†)	16.6 (5.8-50.1)	15.3 (2.2-50.1)	0.630

*Student’s *t*-test.

^†^cde - cumulative dissipated energy.

## DISCUSSION

We found that preoperative treatment with diclofenac significantly decreased the aqueous humor IL-12 concentration and preoperative intraocular inflammation due to diabetic retinopathy and, consequently, reduced the amount of postoperative macular edema caused by phacoemulsification. To the best of our knowledge, this is the first study investigating this effect of an ophthalmic NSAID.

Growing evidence shows that immunological reactions play a major role in the pathogenesis of diabetic retinopathy and that many inflammation mediators may be found in the vitreous body and aqueous humor (9-11). Thus, patients with diabetic retinopathy usually have a subclinical degree of intraocular inflammation, which may worsen the inflammation in cases where cataract surgery is needed. Previous research on postoperative macular edema has focused on its relation to operative manipulation and other possible pathophysiologic mechanisms. However, despite the many studies available, few have addressed the issue of a significantly greater incidence of postoperative macular edema in patients with diabetic retinopathy (14). Analyzing the systematic reviews by Wielders et al (25) and Kessel et al (26) on comparing the effect of topical steroids with topical NSAIDs in controlling inflammation and preventing macular edema after cataract surgery in patients with diabetic retinopathy, we found seven trials reporting that the combination of topical corticosteroids and topical NSAIDs reduced the odds of developing macular edema after cataract surgery as compared to topical corticosteroids as a single-drug treatment. We found only one trial comparing NSAIDs and corticosteroids as a single-drug treatment in patients with non-proliferative diabetic retinopathy, but without preoperative administration of NSAIDs (16). We found two studies comparing NSAIDs started 1 to 3 days before surgery vs on the day of surgery or day after, but unlike our study, these studies enrolled only non-diabetic patients and found that preoperative administration of ketorolac and diclofenac were significantly more effective in controlling inflammation than if the drug administration was started on the day of surgery (20,27). Singh et al (22) evaluated preoperative and postoperative NSAID (nepafenac) ophthalmic solution in the prevention of macular edema following cataract surgery in diabetic retinopathy patients and found significantly lower percentage of patients in the nepafenac group that developed macular edema relative to patients in the sham group.

Pseudophakic cystoid macular edema was 6 to 7 times more prevalent in patients randomized to topical steroids compared with topical NSAIDs when evaluated by fluorescein angiography or OCT at 4 to 5 weeks after cataract surgery (26). Shimura et al (19) and Ching et al (28) found that macular thickness assessed by OCT peaks at approximately 4 to 8 weeks postoperatively. In our study, CFT peaked in the experimental group at 4 weeks after surgery, with subsequent gradual decrease toward the week 12 after surgery, and in the control group, CFT was progressively increasing and peaked at week 12 after surgery, what could be explained by different study populations according to diabetic status.

Singh et al (22) evaluated preoperative and postoperative NSAID (nepafenac) ophthalmic solution in the prevention of macular edema following cataract surgery in diabetic retinopathy patients and they found significantly lower percentage of patients in the nepafenac group who developed macular edema relative to patients in the sham group. Our results also showed that patients treated preoperatively and postoperatively with diclofenac 7 days before phacoemulsification had significantly lower increase in CFT, which suggests that preoperative administration of topical NSAIDs lowers the incidence of postoperative macular edema in patients with diabetic retinopathy. We have also found lower intraocular IL-12 concentration in diclofenac-treated group, which suggests that intraocular inflammation may play a significant role in the macular edema formation.

We found no significant correlation between IL-12 concentration in the aqueous humor and CFT change in the 7-day period before the surgery. One reason may be that the enrolled patients had already had a diabetic retinopathy with consequent macular edema to a different extent. However, IL-12 is not the only inflammatory mediator involved in macular edema formation and further research should address this issue.

The main limitation of this study is the possibly short follow-up time, because our resulted showed that CFT was increasing in the control group up to the last follow-up visit, which was 90 days after the surgery. From our clinical experience, optimal follow-up time would be 12 months, because macular edema after cataract surgery can persist up to one year. Further research should focus on investigating the effectiveness of preoperative NSAID therapy in the treatment and prevention of postoperative macular edema on a larger number of patients with diabetic retinopathy.
